# Seed Germination Enhancement of Two *Balanites* Species (*B. aegyptiaca* (L.) Del. and *B. rotundifolia* (Tiegh.) Blatt.) Using Different Presowing Treatments in Ethiopia

**DOI:** 10.1155/2023/5571489

**Published:** 2023-12-26

**Authors:** Mohammed Adefa Seid, Tigist Wondimu, Asfaw Degu, Awol Assefa

**Affiliations:** Department of Plant Biology and Biodiversity Management, Addis Ababa University, P.O. Box 1176, Addis Ababa, Ethiopia

## Abstract

*B. aegyptiaca* and *B. rotundifolia* are known to be multipurpose trees with various uses and values. Therefore, the aim of this study was to investigate the seed germination behaviours of *B. aegyptiaca* and *B. rotundifolia* under different presowing treatments. Hence, seeds were collected from the Central and Southern Ethiopian Rift Valley regions. Then, a total of 864 fruits (seeds) subjected to eight different presowing treatments and planted in pots arranged in a completely randomized design (CRD) were tested for each species. The mean germination percentage (GP), mean daily germination percentage (GD), mean germination time (GT), and mean germination index (GI) were computed. One-way ANOVA showed the presence of significant GP, GD, GT, and GI among treatment groups at *p* < 0.05 under both *Balanites* species. For *B. aegyptiaca*, Tukey's HSD test showed that seeds soaked with 98% H_2_SO_4_ for 10 minutes (*98HSO10m*) and 20 minutes (*98HSO20m*) have the highest GPs (87 ± 8.8 and 82 ± 10.2, respectively) that are significant at *p* < 0.05. The seeds soaked in 75°C hot water for 10 minutes and subsequently cooled for 12 hours (*HW75d*), *98HSO10m*, and *98HSO20m* have the highest GDs (2%) that are significant at *p* < 0.05. Moreover, *98HSO20m*, *98HSO10m*, and seeds soaked in cold water for 48 hours at room temperature of 25°C (*CW48h*) have the shortest GTs (24 ± 2.2, 25 ± 0.5, and 25 ± 1.3, respectively), and *98HSO10m* and *98HSO20m* have the highest GIs (1.04 ± 0.09 and 1.01 ± 0.08, respectively) that are significant at *p* < 0.05. For *B. rotundifolia*, the control recorded the highest cumulative germination (i.e., 71), followed by CW48h (i.e., 51). However, Tukey's HSD tests generally indicated that no treatment group resulted in significant differences in the means of GP, GD, GT, and GI at *p* < 0.05. So, no treatment group was observed to enhance the germination of *B. rotundifolia* compared to the control. However, this study generally indicated potential seed enhancement technologies for *B. aegyptiaca* with greater implications for propagation, conservation, and sustainable utilization of the species in the agricultural and pastoral communities of Ethiopia.

## 1. Introduction

*B. aegyptiaca* and *B. rotundifolia* are known to be multipurpose trees with various uses and values [1]. The species are widely distributed in arid and semiarid ecosystems in Ethiopia [1–3]. Despite all these products, the species are considered one of the underutilized, neglected arid zone tree species that need to be domesticated [4]. Tree seeds always exhibit some degree of dormancy, resulting in a delay and irregularity in the germination of seeds in the nursery and even on forest floors [5, 6]. The seeds of *Balanites* are generally highly variable among sources in weight and morphology [7], as well as in germination capacity [8, 9]. The seed storage behaviour of *Balanites* is orthodox in which seed viability can be maintained for 2 or even several years in air-dry storage at cool temperatures or hermetic storage at 3°C with 6–10% moisture content [1].

Different seed treatments, such as cold and hot water, were reported to enhance the germination of *B. aegyptiaca* [10]. Fresh seeds of *B. aegyptiaca* are believed to germinate readily without treatments, and the presence of mesocarp is thought to delay the seed germination of *B. aegyptiaca* [9]. The seed storage behaviour of *B. aegyptiaca* is orthodox in which seed viability can be maintained for two or even several years in air-dry storage at cool temperatures or hermetic storage at 3°C with 6–10% moisture contents [1]. The species can be propagated by seedlings (seeds) using direct sowing as well as by applying presowing treatments to maximize the germination percentage [1–3]. Different seed treatments, such as cold and hot water, have been reported to enhance the germination of *Balanites* [10–12]. *B. rotundifolia* is a very drought-resistant tree, even more so than *B. aegyptiaca* [13, 14]. The fruits of *B. rotundifolia* are brittle and smooth on the outside, with a fibrous and oily endocarp with a high moisture content [14]. *B. rotundifolia* is the less-studied *Balanites* in terms of its seed germination behaviour compared to *B. aegyptiaca*. However, direct sowing of the seed (or fruits) without applying treatments has previously been suggested [14].

Effective propagation and seedling establishment are the basic requirements for sustainable management for a species with notable seed dormancy profiles. Indigenous fruit trees play an important role in the livelihood of rural people in sub-Saharan Africa and are sources of nutrition for the local population [15]. Hence, understanding the seed physiology of plants can enhance future conservation through afforestation. Previously, Elfeel [9] studied the germination of Saudi Arabia's *B. aegyptiaca* using water soaking and different sowing orientations. However, extensive studies are yet to be conducted for populations of *B. aegyptiaca* and *B. rotundifolia* in other geographical areas, including Ethiopia. Moreover, previously, no information was documented on the seed germination behaviour of *Balanites* in Ethiopia using the different seed treatments. In this regard, documentation of the seed germination behaviours of species is very important for successful utilization or conservation plans [16]. It was hypothesized that presowing seed treatments could enhance the germination of *B. aegyptiaca* and *B. rotundifolia*. Therefore, the present study was carried out to evaluate the potentiality of different seed presowing treatments for enhanced *B. aegyptiaca* and *B. rotundifolia* germination.

## 2. Materials and Methods

### 2.1. Seeds Sampling

**Figure 1:**
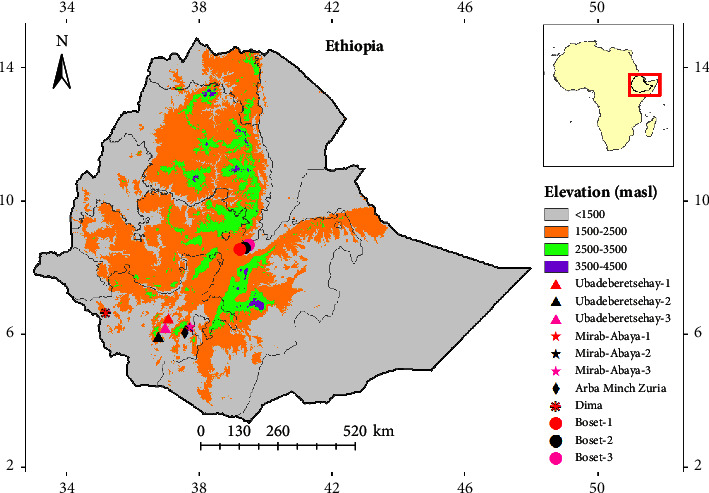
Seed collection sites of *B. aegyptiaca* and *B. rotundifolia* in Ethiopia.

The seeds were mainly collected from the Central and Southern Ethiopian Rift Valley regions (Figure 1). The seeds collection areas mainly included the arid and semiarid ecosystems that include the Acacia-Commiphora woodland and bushland proper, *Acacia* wooded grassland of the Rift Valley, and Combretum-Terminalia woodland and wooded grassland ecosystems of Ethiopia [17].

Seeds collections were done from February to November 2022, following the previous observations from specimen sampling for morphological study and phenetic analysis of the *Balanites* genus in the study area. Hence, the seeds of *B. aegyptiaca* were collected from Boset district-1, 2, and 3 in Oromia region (i.e., 39.21644°E, 8.54042°N; 39.35609E, 8.59369°N; 39.4755°E, 8.6767°N), Ubadeberetsehay district-1 (i.e., 37.06422°E, 6.47314°N) and Arba Minch Zuria district (i.e., 37.56822°E, 6.036998°N) in Southern Nations, Nationalities, and Peoples Region (SNNPR), and Dima district (i.e., 35.18337°E, 6.64147°N) in Gambella region; *B. rotundifolia* from Mirab-Abaya district-1, 2, and 3 (i.e., 37.697543°E, 6.181298°N; 37.710982°E, 6.199166°N; 37.732482°E, 6.232607°N) and Ubadeberetsehay district-2 and 3 (i.e., 36.76659°E, 5.92465°N; 36.9647°E, 6.19399°N) in SNNPR.

The seeds were collected from conspicuous superior (mother) trees of *B. aegyptiaca* and *B. rotundifolia*. So, about 30–40 fruits (seeds) per mother tree (i.e., about 200 fruits (seeds) per population) were collected and mixed up to represent the population. In other words, the fruits (seeds) from different populations (provenances) were collected from different parts of Ethiopia and thoroughly mixed up and homogenized to represent the species. The homogenized seeds were further processed, cleaned, and purified from inert and unwanted materials.

**Figure 2:**
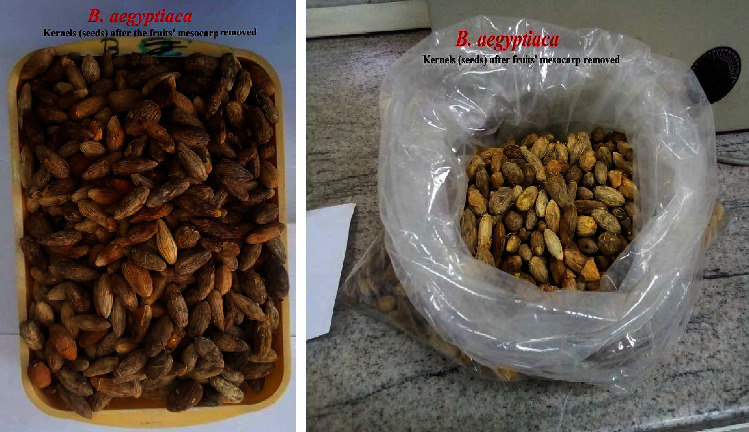
Kernels (seeds) of *B. aegyptiaca* after the fruits' mesocarps are removed.

**Figure 3:**
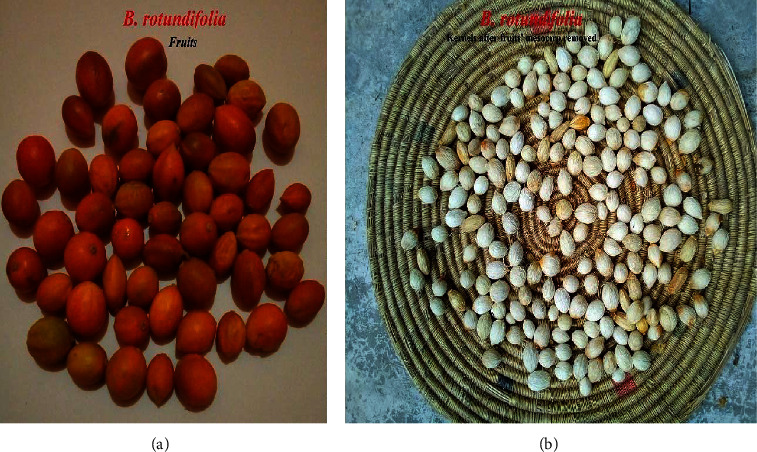
Fruits (a) and kernels (seeds) of *B. rotundifolia* after the fruits' mesocarps are removed (b).

The mesocarps, i.e., the fleshy and edible parts of the fruit, were also removed (Figures 2 and 3). The seeds were dried, and the moisture content (MC) was adjusted to 6.0% (checked with a German-made KERN-DBS 60-3 moisture tester).

### 2.2. Preparations of Seed Treatments and Experimental Layout

For this purpose of study, seed treatments were prepared based on literature and ISTA protocols [1, 9, 10, 18–20]. Accordingly, the seven seed treatments (excluding the control) were prepared (Table 1). The International Seed Testing Association (ISTA) recommends four replicates of 100 seeds each for standard germination tests, but there should not be fewer than 100 seeds in replicates of 25 or 50 seeds [21–23].

**Figure 4:**
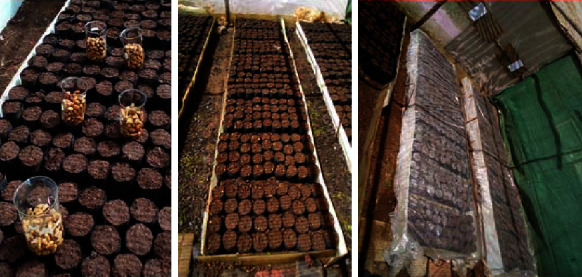
The nursery beds and the arrangement of pots, sown seeds, and sown seeds covered with transparent polythene sheet.

Following the random table of Gomez and Gomez [24], the experiment was laid out in a completely randomized design (CRD) with eight treatments and four replications each. Hence, 27 seeds per replication (i.e., 9 pots per replication, 3 seeds per pot, 108 seeds per treatment, and 864 fruits (seeds) for 8 treatments) were tested. The germinations were done in a standard nursery soil mixture (3 : 2 : 1 ratio of topsoil, manure, and sand) placed in polyethylene plastic pots with a 16 cm diameter (Figure 4). Generally, *B. aegyptiaca has fruits with* lengths ranging from 2 to 4 cm and widths of 1.5 to 2.5 cm, while *B. rotundifolia* has fruits with lengths ranging from 2 to 3 cm and widths of 2 to 2.5 cm. So, fruits (seeds) were sown in each pot with a horizontal orientation of the seed stalk while fully covering the fruits (seeds) with the substrate (∼2.5 cm depth). The sown seeds (or fruits) were then covered with transparent polythene sheets to optimize and regulate the moisture and temperature.

The germination counts were taken every other day, starting one week after sowing, for three months, after which no more germination was observed in two weeks' time. In this study, a seed was considered to have germinated when the radicle emerged conspicuously [25, 26]. All experimental activities were carried out in the nursery house of the Central Ethiopia Forestry Development Center (CEFDC)-Ethiopian Forestry Development (EFD), Addis Ababa. Over the course of the germination test, the morning average temperature was 20°C, the midday average was 26.5°C, and the evening average was 19.5°C.

### 2.3. Statistical Data Analysis

The data analysis was based on untransformed seed germination percentages. The dataset was checked with a histogram and has a normal distribution. The nineteen germination measurements (or parameters) were generated (see Supplementary Tables 1 and 2), of which four parameters, namely, the mean germination percentage (GP), mean daily germination percentage (GD), mean germination time (GT), and mean germination index (GI), were analysed and presented for this study report. The GP, which is an estimate of the germinability of the population of seeds, was calculated as follows:

(1) $$\begin{array}{c} \mathrm{GP}=\frac{\sum_{i=1}^{k}ni}{N}x\;100, \end{array}$$

where *n*_*i*_ is the number of seeds germinated in the *i*^th^ time and *N* is the total number of seeds used.

The GD, which is the mean number of seeds germinated per day (i.e., the number of seeds germinated daily relative to the maximum number of germinated seeds), was also calculated using the following Adams and Farrish [27]:

(2) $$\begin{array}{c} \mathrm{GD}=\left(\frac{\mathrm{CP}}{\text{Tn}}\right), \end{array}$$

where CP is the final cumulative germination percentage and *T*_*n*_T is the total number of intervals required for final germination.

Following Ellis and Roberts [28], the GT, a measure of the rate and time spread of the germination, of each treatment was calculated using the formula:

(3) $$\begin{array}{c} \mathrm{GT}=\frac{\sum \left(n\;x\;d\right)}{N}, \end{array}$$

where “*n*” is the number of seeds germinated on each day, “*d*” is the number of days from the beginning of the test, and “N” is the total number of seeds germinated at the termination of the experiment.

Furthermore, the GI, which is a measure of the percentage and speed of germination, was calculated using the following formula [29]:

(4) $$\begin{array}{c} \text{GI}=\left(\frac{no.\text{ of germinating seeds}}{\text{days of first count}}\right)+\ldots +\left(\frac{no.\text{ of germinating seeds}}{\text{days of final count}}\right). \end{array}$$

The higher values for this measure indicate a greater rate of germination [30].

Moreover, a one-way ANOVA was computed to see the level of significant difference among the treatment means for the germination parameters at *p* ≤ 0.05. Moreover, Tukey's Honestly Significant Difference (HSD) was computed to assess the significance of the difference between pairs of treatments at *p* ≤ 0.05 [31], allowing all possible pairwise comparisons while keeping the family-wise error rate low.

## 3. Results

### 3.1. Germination of *B. aegyptiaca*

**Figure 5:**
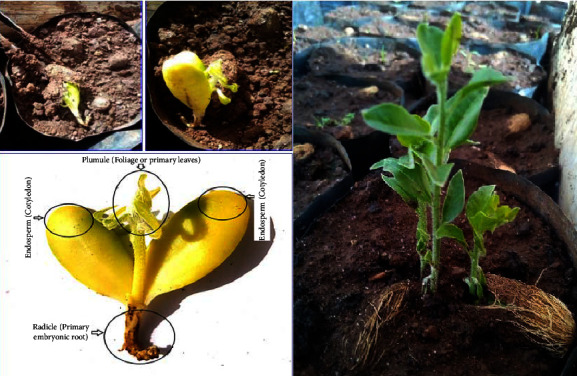
Illustration of newly emerging germinate and fully grown seedlings of *B. aegyptiaca*.

**Figure 6:**
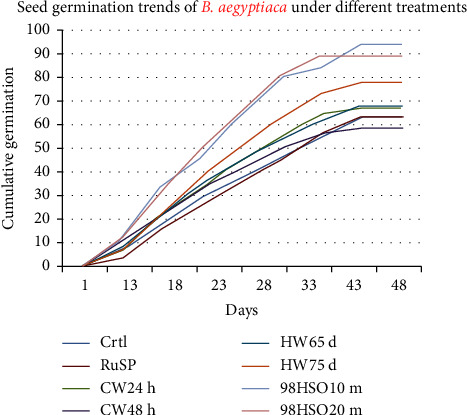
Cumulative germination of *B. aegyptiaca* under different treatments over days; *Ctrl*: control, RuSP: rubbed with sandpaper, *CW24h*: soaked in cold water for 24 hours at room temperature of 25°C, *CW48h*: soaked in cold water for 48 hours at room temperature of 25°C, *HW65d*: soaked in hot water at 65°C for 10 minutes and left to cool for 12 hours at room temperature of 25°C, *HW75d*: soaked in hot water at 75°C for 10 minutes and left to cool for 12 hours at room temperature of 25°C, *98HSO10m*: soaked in 98% H_2_SO_4_ for 10 minutes and subsequent rinsed with water, and *98HSO20m*: soaked in 98% H_2_SO_4_ for 20 minutes and subsequent rinsed with water.

Figure 5 illustrates the newly emerging germinate and fully grown seedlings of *B. aegyptiaca*. After the six weeks of the germination period, no new germinates were observed in most of the treatments (Figure 6).

The highest cumulative germination percentage (94%) was recorded by seeds soaked in 98% H_2_SO_4_ for 10 minutes and subsequent rinsed with water (*98HSO10m*), followed by seeds soaked in 98% H_2_SO_4_ for 20 minutes and subsequent rinsed with water (*98HSO20m*) with 89% cumulative germination percentage, and seeds soaked in hot water at 75°C for 10 minutes, and left to cool for 12 hours at room temperature of 25°C (*HW75d*) with 78% cumulative germination percentage. The lowest cumulative germination percentage was recorded by seeds soaked in cold water for 48 hours at room temperature of 25°C (*CW48h*) with a 58% cumulative germination percentage (Figure 6).

The one-way ANOVA also indicated that there exist significant mean variations among treatment groups for the different germination parameters (Table 2). Tukey's HSD test also showed that *98HSO10m* (87 ± 8.8) and *98HSO20m* (82 ± 10.2) have the highest GPs which are significant at *p* < 0.05 compared to the other treatment groups. Moreover, Tukey's HSD indicated that *HW75d*, *98HSO10m*, and *98HSO20m* have the highest GDs (2%) that are significant at *p* < 0.05 compared to the other treatment groups (Table 3). Tukey's HSD for GT also showed that *98HSO20m* (24 ± 2.2), *98HSO10m* (25 ± 0.5) and *CW48h* (25 ± 1.3) have the shortest GTs that are significant at *p* < 0.05 compared to the other treatment groups (Table 3). Most importantly, Tukey's HSD resulted that *98HSO10m* (1.04 ± 0.09) and *98HSO20m* (1.01 ± 0.08) have the highest GIs that are significant *p* < 0.05 compared to the other treatment groups (Table 3).

### 3.2. Germination of *B. rotundifolia*

**Figure 7:**
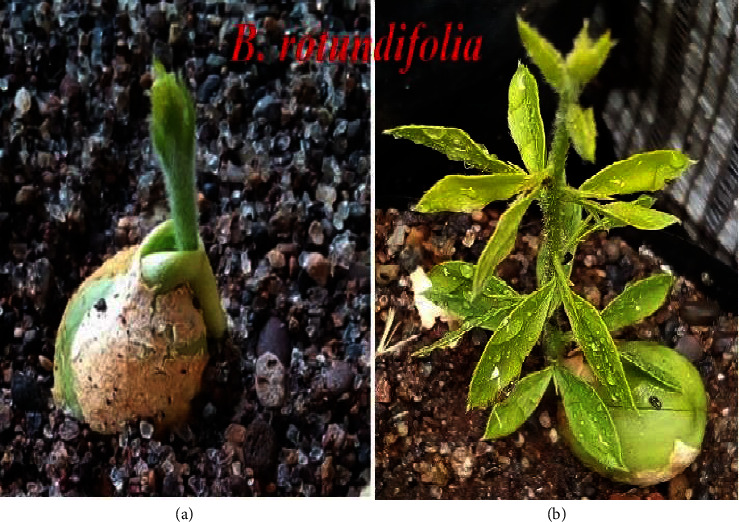
A newly emerging germinate (a) and fully grown seedlings of *B. rotundifolia* (b).

**Figure 8:**
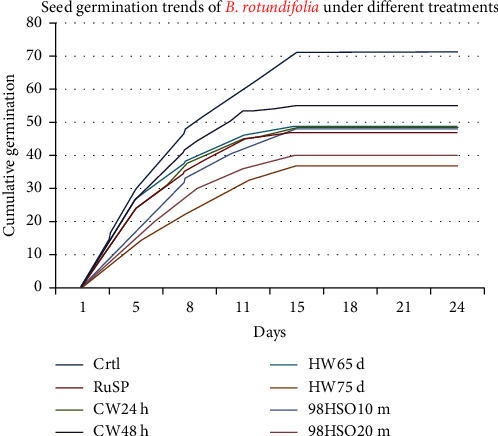
Cumulative germination of *B. rotundifolia* under different treatments over days; *Ctrl*: control, RuSP: rubbed with sandpaper, *CW24h*: soaked in cold water for 24 hours at room temperature of 25°C, *CW48h*: soaked in cold water for 48 hours at room temperature of 25°C, *HW65d*: soaked in hot water at 65°C for 10 minutes and left to cool for 12 hours at room temperature of 25°C, *HW75d*: soaked in hot water at 75°C for 10 minutes and left to cool for 12 hours at room temperature of 25°C, *98HSO10m*: soaked in 98% H_2_SO_4_ for 10 minutes and subsequent rinsed with water, and *98HSO20m*: soaked in 98% H_2_SO_4_ for 20 minutes and subsequent rinsed with water.

This study generally demonstrated that *B. rotundifolia* germinates rapidly in a couple of weeks.  Figure 7 illustrates the newly emerging germinate and fully grown seedlings of *B. rotundifolia*. In this regard, it was observed that the seeds of *B. rotundifolia* have shorter and faster germination time trends compared to those of *B. aegyptiaca* (Figure 8).

Hence, the highest cumulative germination was recorded by the *Ctrl* (i.e., 71), followed by *CW48h* (i.e., 51) (Figure 8). The one-way ANOVA also indicated that there exist significant mean variations among treatment groups for the different germination parameters (Table 4). However, Tukey's HSD test showed that the control has the highest GP (66 ± 10.2) that is significant at *p* < 0.05 compared to the other treatment groups (Table 5), while the lowest significant GP at *p* < 0.05 was observed by *HW75d* (34% ± 7.0).

Overall, except for *HW75d* and *98HSO20m*, the GDs of the different treatments were found to be 2 germination parentages per day that are significant at *p* < 0.05. The species tend to have a mean germination time (GT) of less than 10 days regardless of the treatment type applied. Tukey's HSD test for GT indicated that there exist no significant mean differences among treatment groups, except *HW75d*, at *p* < 0.05. Tukey's HSD test for GI also showed that the control has not significant different GI compared to the remaining treatment groups, except *HW75d* and *98HSO20m*, at *p* < 0.05.

## 4. Discussion

In the current study, some presowing treatment techniques enhanced the germination capability of the seeds of *B. aegyptiaca* compared to seeds receiving no prior presowing treatment (i.e., the control). Generally, soaking the fruits (seeds) in 98% H_2_SO_4_ and water (hot or cold) enhances the germination percentages of *B. aegyptiaca*. In particular, seeds soaked with 98% H_2_SO_4_ for 10 and 20 minutes effectively broken the seeds dormancy and significantly improved the mean germination percentages (GP). The soaking of fruits (seeds) with hot water for up to 20 minutes has also a promising effect on the germination of *B. aegyptiaca*. The fibrous endocarp of fruits needs to be digested to increase the inlet of moisture and oxygen to the embryo for germination to start. A study by Ahmed et al. [32] reported that the highest germination percentage (>80%) was observed for seeds of *B. aegyptiaca* from Saudi Arabia soaked with cold distilled water for 24 hours. Similarly, a study by Elfeel [9] in Saudi Arabia also reported that seeds soaked with cold water for 18 and 24 hours resulted in a higher germination percentage compared to seeds soaked for 48 hours. Similarly, soaking the seed in cold water for 24 hours is recommended by Bekele-Tesemma [2, 3], and a germination rate of 50–70% can be attained. The observation of such variations in seed germination is likely due to the variability of the physical, physiological, and genetic quality of the seeds of the different populations of *B. aegyptiaca* distributed in wider ranges of ecological conditions. Therefore, the physical, physiological, and genetic differences among populations greatly affect their rate of water and chemical uptake by the seeds [33, 34].

Seed soaking duration affects embryonic physiological activities toward germination and ultimately determines the seed germination percentage, both negatively and positively [35, 36]. Increasing the soaking duration in both cold and hot water is very likely to decrease the germination percentage of *B. aegyptiaca* [9, 32], which was also observed in our present study. Similarly, prolonged soaking of seeds in highly concentrated H_2_SO_4_ could cause damage in both the external and internal parts of the seeds, which ultimately damages the embryonic tissue, resulting in lower germination percentages [29, 37]. Overall, the optimal seed soaking duration required for attaining a better germination percentage varies with the species and the ecological conditions under which it evolved. Furthermore, apart from presowing treatments, the sowing orientations of seeds in the soil substrate also affect the germination percentages. For instance, for *B. aegyptiaca*, the horizontal orientation of the seed stalk was recommended to attain higher germination [9], while Hall and Walker [10] recommend the seed be sown vertically with the stalk end downwards.

In this study, seeds treated with 98% H_2_SO_4_ were observed to reduce the germination time required for the seed populations to complete their germination. The shorter the germination time, the shorter the nursery period, and the faster the species reach for plantation and forestry work. In the contrary, a study by Elfeel [9] reported that seeds soaked with water for 18 and 24 hours, as well as untreated seeds, resulted in the shortest germination time compared to seeds soaked with water for 48 hours. According to Hall and Walker [10] and Bekele-Tesemma [3], the germination of *B. aegyptiaca* generally occurs in 1–4 weeks, which also agrees with our present study. Overall, the current study indicated the potential of seed presowing treatment technologies to enhance seed germination in *B. aegyptiaca*, which has greater implications for the conservation and sustainable utilization of the species in the semiarid ecosystems of Ethiopia and beyond.

The germination of *B. rotundifolia*, however, was not enhanced by any treatment applied. This might be due to the morphology of the fruit and germplasm of *B. rotundifolia*, which has softer fruits and seed germplasm that are very likely injured by the treatments used, either mechanical or biochemical ones. However, for *B. rotundifolia*, having a good germination percentage (>70%) without treatments is very advantageous in terms of propagation and conservation strategies. Seed propagation without treatment application is by far the most cost-effective approach. The shorter germination time and faster germination rate observed by *B. rotundifolia* are the two additional advantages of the species for easy propagation and cultivation. Therefore, the application of treatments to the fruit (seed) of *B. rotundifolia* is not necessary [14]. The fruits of *B. rotundifolia* are brittle and smooth on the outside, with a fibrous and oily endocarp and high moisture content. This probably helped the species maintain the sufficient moisture required for germinating in dryland. That is why the species is a very drought-resistant tree, even more so than *B. aegyptiaca* [13, 14].

## 5. Conclusions

This study identified the potentiality of presowing seed treatment as a tool for enhancing the seeds germination of *B. aegyptiaca*. In general, soaking of seeds in 98% concentrated H_2_SO_4_, hot water as well as in normal cold water has proven to be effective methods to break the seed dormancy, improving the germination percentages, and reducing the mean germination time required to complete the germination. Yet, the untreated seeds of *B. aegyptiaca* also exhibited satisfactory germination (>50%), with implications of the high degree of regeneration and subsequent ecological succession of the species under the natural conditions of recurrent climate change and habitat fragmentation. However, no presowing treatments applied enhanced the seeds germination of *B. rotundifolia*, where more than 70% of seed germination was attained without the application of treatments. This has an implication of the high degree of regeneration and subsequent ecological succession of *B. rotundifolia* under the natural conditions of recurrent climate change and habitat fragmentation. Therefore, both species can be generally considered as alternatives for dryland rehabilitation and forestry development. Overall, these findings will contribute to the conservation and sustainable utilization of the species in the agricultural and pastoral communities of Ethiopia.

## Figures and Tables

**Table 1:** Presowing treatments designed to study the seed germination of *B. aegyptiaca* and *B. rotundifolia*.

No.	Seed treatment types
1	Control, i.e., received no prior seed treatment (*Ctrl*)
2	Rubbed with sandpaper (*RuSP*)
3	Soaked with cold normal water for 24 hours at room temperature of 25°C (*CW24h*)
4	Soaked with cold normal water for 48 hours at room temperature of 25°C (*CW48h*)
5	Soaking with hot normal water at 65°C for 10 minutes, and left to cool for 12 hours at room temperature of 25°C (*HW65d*)
6	Soaked with hot normal water at 75°C for 10 minutes, and left to cool for 12 hours at room temperature of 25°C (*HW75d*)
7	Soaked with 98% H_2_SO_4_ for 10 minutes and subsequent rinsed with water (*98HSO10m*)
8	Soaked with 98% H_2_SO_4_ for 20 minutes and subsequent rinsed with water (*98HSO20m*)

**Table 2:** One-way ANOVA of the mean significance difference among treatment groups for their effect on mean germination percentage (GP), daily germination percentage (GD), germination time (GT), and germination index (GI) of *B. aegyptiaca*.

	Df	Sum square	Mean square	*F* value	Pr (>F)
*GP*					
Treatments	7	4162	594.6	10.52	5.38*e* − 06^*∗∗∗*^
Residuals	24	1356	56.5		

*GD*					
Treatments	7	1.8053	0.25791	10.59	5.09*e* − 06^*∗∗∗*^
Residuals	24	0.5846	0.02436		

*GT*					
Treatments	7	60.72	8.674	3.429	0.011^*∗*^
Residuals	24	60.72	2.530		

*GI*					
Treatments	7	0.7313	0.10447	16.27	1.05*e* − 07^*∗∗∗*^
Residuals	24	0.1541	0.00642		

Significance codes: ^*∗∗∗*^highly significant if *p* ≤ 0.001, ^*∗∗*^very significant if *p* ≤ 0.01, and ^*∗*^significant if *p* ≤ 0.05.

**Table 3:** Summary of GP, GD, GT, GI, standard deviation (SD), and standard error (SE) of the germination parameters of *B. aegyptiaca*.

Treatments	GP ± SD (%)	SE	GD ± SD (%)	SE	GT ± SD (days)	SE	GI ± SD (%/day)	SE
*Ctrl*	58 ± 1.9^a^	0.9	1 ± 0.0^a^	0.0	28 ± 2.5^a^	1.3	0.65 ± 0.06^a^	0.0
*RuSP*	58 ± 6.3^a^	3.2	1 ± 0.1^a^	0.1	28 ± 1.5^a^	0.7	0.62 ± 0.05^a^	0.0
*CW24h*	62 ± 6.3^a^	3.2	1 ± 0.1^a^	0.1	26 ± 1.7^a^	0.8	0.74 ± 0.06^a^	0.0
*CW48h*	54 ± 6.4^a^	3.2	1 ± 0.1^a^	0.1	25 ± 1.3^b^	0.6	0.67 ± 0.07^a^	0.0
*HW65d*	63 ± 10.0^a^	5.0	1 ± 0.2^a^	0.1	26 ± 0.9^a^	0.5	0.73 ± 0.14^a^	0.1
*HW75d*	72 ± 6.4^a^	3.2	2 ± 0.1^b^	0.1	27 ± 1.0^a^	0.5	0.82 ± 0.07^a^	0.0
*98HSO10m*	87 ± 8.8^b^	4.4	2 ± 0.2^b^	0.1	25 ± 0.5^b^	0.3	1.04 ± 0.09^b^	0.0
*98HSO20m*	82 ± 10.2^b^	5.1	2 ± 0.2^b^	0.1	24 ± 2.2^b^	1.1	1.01 ± 0.08^b^	0.0

The significance difference between pairs of treatments at *p* ≤ 0.05 is calculated using Tukey's HSD test. The values denoted with the same alphabet(s) along the vertical column of each germination parameter are not significantly different at *p* ≤ 0.05, and vice versa.

**Table 4:** One-way ANOVA of the mean significance difference among treatment groups for their effect on mean germination percentage (GP), daily germination percentage (GD), germination time (GT), and germination index (GI) of *B. rotundifolia*.

	Df	Sum square	Mean square	*F* value	Pr (>F)
*GP*					
Treatments	7	2602	371.8	4.144	0.00406^*∗∗*^
Residuals	24	2153	89.7		

*GD*					
Treatments	7	3.528	0.5039	4.105	0.00428^*∗∗*^
Residuals	24	2.946	0.1228		

*GT*					
Treatments	7	9.053	1.2934	5.271	0.000957^*∗∗∗*^
Residuals	24	5.889	0.2454		

*GI*					
Treatments	7	4.446	0.6351	4.303	0.00328^*∗∗*^
Residuals	24	3.542	0.1476		

Significance codes: ^*∗∗∗*^highly significant if *p* ≤ 0.001, ^*∗∗*^very significant if *p* ≤ 0.01, and ^*∗*^significant if *p* ≤ 0.05.

**Table 5:** Summary of GP, GD, GT, GI, standard deviation (SD), and standard error (SE) of the germination parameters of *B. rotundifolia*.

Treatments	GP ± SD (%)	SE	GD ± SD (%)	SE	GT ± SD (days)	SE	GI ± SD (%/day)	SE
*Ctrl*	66 ± 10.2^a^	5.1	2 ± 0.4^a^	0.2	8.3 ± 0.5^a^	0.2	2.53 ± 0.46^a^	0.2
*RuSP*	44 ± 4.7^b^	2.3	2 ± 0.2^a^	0.1	7.4 ± 0.6^a^	0.3	1.81 ± 0.28^ab^	0.1
*CW24h*	44 ± 9.1^b^	4.5	2 ± 0.3^a^	0.2	7.3 ± 1.1^a^	0.5	1.83 ± 0.28^ab^	0.1
*CW48h*	51 ± 14.0^b^	7.0	2 ± 05^a^	0.3	7.3 ± 0.1^a^	0.1	2.11 ± 0.57^ab^	0.3
*HW65d*	45 ± 1.9^b^	0.9	2 ± 0.1^a^	0.0	7.2 ± 0.2^a^	0.1	1.93 ± 0.11^ab^	0.1
*HW75d*	34 ± 7.0^c^	3.5	1 ± 0.3^b^	0.1	8.6 ± 0.2^b^	0.1	1.25 ± 0.23^b^	0.1
*98HSO10m*	44 ± 15.1^b^	7.6	2 ± 0.6^a^	0.3	8.4 ± 0.2^a^	0.1	1.66 ± 0.59^ab^	0.3
*98HSO20m*	37 ± 5.2^b^	2.6	1 ± 0.2^b^	0.1	8.1 ± 0.4^a^	0.2	1.41 ± 0.26^b^	0.1

The significance difference between pairs of treatments at *p* ≤ 0.05 is calculated using Tukey's HSD test. The values denoted with the same alphabet(s) along the vertical column of each germination parameter are not significantly different at *p* ≤ 0.05, and vice versa.

## Data Availability

The data used to support the findings of this study are available from the corresponding author upon reasonable request.

## Supplementary Materials

Supplementary Materials Supplementary Table 1. Germination measurements (metadata) of *B. aegyptiaca* under different presowing treatments. Supplementary Table 2. Germination measurements (metadata) of *B. rotundifolia* under different presowing treatments.
