# Supplementary material for: Seed Germination Enhancement of Two Balanites Species (B. aegyptiaca (L.) Del. and B. rotundifolia (Tiegh.) Blatt.) Using Different Presowing Treatments in Ethiopia
Source: Scientifica (Cairo). 2023 Dec 26;2023:5571489. doi: 10.1155/2023/5571489 (PMC10761227; doi:10.1155/2023/5571489)
Supplement: Supplementary Materials — Supplementary Table 1. Germination measurements (metadata) of B. aegyptiaca under different presowing treatments. Supplementary Table 2. Germination measurements (metadata) of B. rotundifolia under different presowing treatments. [file 5571489.f1.zip › Supplementary table 1 (1).pdf]

Supplementary table 1. Germination measurements (metadata) of *B. aegyptiaca* under different pre-sowing treatments; *Ctrl* = control, *RuSP* = Rubbed with sandpaper, *CW24h* = Soaked in cold water for 24 hours at room temperature of 25°C, *CW48h* = Soaked in cold water for 48 hours at room temperature of 25°C, *HW65d* = Soaked in hot water at 65°C for 10 minutes, and left to cool for 12 hours at room temperature of 25°C , *HW75d* = Soaked in hot water at 75°C for 10 minutes, and left to cool for 12 hours at room temperature of 25°C, *98HSO10m* = Soaked in 98% H_2SO_4 for 10 minutes and subsequent rinsed with water, and *98HSO20m* = Soaked in 98% H_2SO_4 for 20 minutes and subsequent rinsed with water.

Factor	GP	R	MGT	MGR	CV_t	CVG	GI	U	Z	T_{10}	T_{25}	T_{50}	T_{75}	T_{90}	T_{10-90}	T_{25-75}	MDG	Peak value	G value
Ctrl	59	64	27.06	0.037	37.09	3.695	0.68	2.78	0.09	10.60	16.33	24.00	33.00	39.00	28.40	16.67	1.23	1.38	1.70
Ctrl	59	64	31.44	0.032	27.68	3.181	0.55	2.56	0.12	17.00	21.33	29.67	36.33	40.33	23.33	15.00	1.23	1.38	1.70
Ctrl	59	64	27.06	0.037	37.09	3.695	0.68	2.78	0.09	10.60	16.33	24.00	33.00	39.00	28.40	16.67	1.23	1.38	1.70
Ctrl	56	60	25.67	0.039	38.16	3.896	0.68	2.74	0.10	7.00	14.88	22.17	31.13	36.75	29.75	16.25	1.16	1.36	1.58
RuSP	67	72	30.50	0.033	28.81	3.279	0.64	2.58	0.12	16.00	20.50	28.00	35.50	40.00	24.00	15.00	1.39	1.55	2.15
RuSP	56	60	28.00	0.036	32.37	3.571	0.60	2.69	0.10	13.83	17.58	25.50	33.42	37.17	23.33	15.83	1.16	1.36	1.58
RuSP	52	56	28.00	0.036	33.59	3.571	0.56	2.70	0.10	13.67	17.17	25.50	33.83	37.33	23.67	16.67	1.08	1.27	1.37
RuSP	59	64	27.06	0.037	37.09	3.695	0.68	2.78	0.09	10.60	16.33	24.00	33.00	39.00	28.40	16.67	1.23	1.38	1.70
CW24h	59	64	24.25	0.041	34.91	4.124	0.75	2.56	0.12	7.40	14.67	21.33	29.00	34.00	26.60	14.33	1.23	1.59	1.96
CW24h	63	68	24.76	0.040	34.19	4.038	0.78	2.57	0.12	7.80	15.08	22.17	29.25	33.75	25.95	14.17	1.31	1.68	2.21
CW24h	70	76	28.00	0.036	34.19	3.571	0.77	2.79	0.10	12.40	17.58	25.50	33.42	38.25	25.85	15.83	1.47	1.66	2.43
CW24h	56	60	26.33	0.038	35.64	3.797	0.65	2.74	0.10	10.00	15.92	23.83	31.13	36.75	26.75	15.21	1.16	1.36	1.58
CW48h	63	68	26.24	0.038	39.26	3.812	0.76	2.78	0.10	7.80	15.08	22.17	32.38	38.75	30.95	17.29	1.31	1.46	1.92
CW48h	52	56	23.36	0.043	35.09	4.281	0.68	2.50	0.12	6.60	13.83	20.50	27.17	32.00	25.40	13.33	1.08	1.46	1.58
CW48h	48	52	23.77	0.042	33.09	4.207	0.61	2.51	0.12	8.80	15.08	20.50	27.38	32.25	23.45	12.29	1.00	1.35	1.35
CW48h	52	56	24.79	0.040	34.13	4.035	0.64	2.56	0.11	9.40	15.50	21.33	29.25	34.50	25.10	13.75	1.08	1.36	1.47
HW65d	59	64	27.06	0.037	37.09	3.695	0.68	2.78	0.09	10.60	16.33	24.00	33.00	39.00	28.40	16.67	1.23	1.38	1.70
HW65d	56	60	26.00	0.038	36.20	3.846	0.66	2.74	0.10	10.00	15.92	22.17	31.13	36.75	26.75	15.21	1.16	1.36	1.58
HW65d	59	64	27.38	0.037	35.21	3.653	0.66	2.70	0.11	13.75	16.75	24.00	33.00	39.00	25.25	16.25	1.23	1.38	1.70
HW65d	78	84	25.38	0.039	34.47	3.940	0.94	2.70	0.12	9.40	15.81	22.38	29.25	35.25	25.85	13.44	1.62	2.02	3.27
HW75d	81	88	27.32	0.037	29.51	3.661	0.88	2.62	0.13	14.50	18.50	24.25	31.13	36.00	21.50	12.63	1.70	2.05	3.47
HW75d	70	76	25.89	0.039	33.64	3.862	0.82	2.69	0.12	12.40	16.44	22.38	30.08	35.75	23.35	13.65	1.47	1.80	2.63
HW75d	70	76	25.63	0.039	36.99	3.901	0.85	2.72	0.11	8.60	15.19	22.17	31.13	36.50	27.90	15.94	1.47	1.75	2.57
HW75d	67	72	27.72	0.036	33.01	3.607	0.73	2.71	0.11	14.00	17.38	24.67	32.17	38.50	24.50	14.79	1.39	1.57	2.18
98HSO10m	74	80	24.50	0.041	32.51	4.082	0.91	2.55	0.13	9.00	15.50	21.75	28.71	34.00	25.00	13.21	1.54	2.02	3.12
98HSO10m	93	100	25.60	0.039	32.45	3.906	1.10	2.56	0.14	11.00	16.25	22.50	30.19	34.88	23.88	13.94	1.93	2.44	4.70
98HSO10m	89	96	25.50	0.039	33.71	3.922	1.06	2.54	0.14	10.60	15.50	22.29	30.50	35.00	24.40	15.00	1.85	2.34	4.33
98HSO10m	93	100	25.60	0.039	32.93	3.906	1.10	2.54	0.14	11.00	15.71	23.50	30.19	34.88	23.88	14.48	1.93	2.44	4.70

98HSO20m	89	96	25.71	0.039	29.79	3.890	1.03	2.52	0.14	13.40	17.00	23.00	29.25	34.00	20.60	12.25	1.85	2.36	4.36
98HSO20m	93	100	25.20	0.040	31.41	3.968	1.10	2.55	0.14	11.00	16.25	22.50	28.94	33.83	22.83	12.69	1.93	2.47	4.76
98HSO20m	78	84	25.14	0.040	30.53	3.977	0.92	2.51	0.14	13.10	16.25	22.38	28.94	32.88	19.78	12.69	1.62	2.13	3.46
98HSO20m	70	76	20.89	0.048	32.22	4.786	1.00	2.25	0.18	5.56	12.40	17.50	23.42	28.25	22.69	11.02	1.47	2.25	3.31

Key

**Parameter**	**Parameter description**	**Unit**
GP	Germination percentage	%
R	Relativized percentage	%
MGT	Mean germination time	day
MGR	Mean germination rate	day^{-1}
CV_t	Coefficient of variation of germination time	%, seed day^{-1}
CVG	Coefficient of velocity of germination	%
GI	Germination index	day
U	Uncertainity of germination process	bit
Z	Synchronization index	unit less
T_{10}	Time to 10% germination	day or hour
T_{25}	Time to 25% germination	day or hour
T_{50}	Time to 50% germination	day or hour
T_{75}	Time to 75% germination	day or hour
T_{90}	Time to 90% germination	day or hour
T_{10-90}	Time from 10 to 90% germination	day or hour
T_{25-75}	Time from 25 to 75% germination	day or hour
MGD	Mean daily germination Percent	%
Peak value	Peak value for germination	day^{-1} or $hour^{-1}$
G value	Germination value	
