# Supplementary material for: Seed Germination Enhancement of Two Balanites Species (B. aegyptiaca (L.) Del. and B. rotundifolia (Tiegh.) Blatt.) Using Different Presowing Treatments in Ethiopia
Source: Scientifica (Cairo). 2023 Dec 26;2023:5571489. doi: 10.1155/2023/5571489 (PMC10761227; doi:10.1155/2023/5571489)
Supplement: Supplementary Materials — Supplementary Table 1. Germination measurements (metadata) of B. aegyptiaca under different presowing treatments. Supplementary Table 2. Germination measurements (metadata) of B. rotundifolia under different presowing treatments. [file 5571489.f1.zip › Supplementary table 2 (1).pdf]

Supplementary table 2. Germination measurements (metadata) of *B. rotundifolia* under different pre-sowing treatments; *Ctrl* = control, *RuSP* = Rubbed with sandpaper, *CW24h* = Soaked in cold water for 24 hours at room temperature of 25°C, *CW48h* = Soaked in cold water for 48 hours at room temperature of 25°C, *HW65d* = Soaked in hot water at 65°C for 10 minutes, and left to cool for 12 hours at room temperature of 25°C , *HW75d* = Soaked in hot water at 75°C for 10 minutes, and left to cool for 12 hours at room temperature of 25°C, *98HSO10m* = Soaked in 98% H_2SO_4 for 10 minutes and subsequent rinsed with water, and *98HSO20m* = Soaked in 98% H_2SO_4 for 20 minutes and subsequent rinsed with water.

Factor	GP	R	MGT	MGR	CV_t	CVG	GI	U	Z	T_{10}	T_{25}	T_{50}	T_{75}	T_{90}	T_{10-90}	T_{25-75}	MDG	Peak value	G value
Ctrl	70	90	8.16	0.123	46.09	12.258	2.77	1.82	0.28	1.84	3.11	5.38	9.25	12.47	10.62	6.14	2.61	6.67	17.38
Ctrl	56	71	9.00	0.111	42.41	11.111	1.97	1.97	0.21	2.20	4.00	6.88	10.25	13.00	10.80	6.25	2.06	4.17	8.57
Ctrl	59	76	7.94	0.126	43.51	12.598	2.34	1.80	0.28	1.91	3.29	5.60	9.29	11.80	9.89	6.00	2.19	5.56	12.19
Ctrl	78	100	8.14	0.123	43.81	12.281	3.02	1.84	0.27	1.93	3.33	5.75	8.75	12.20	10.27	5.42	2.88	6.94	20.00
RuSP	48	62	7.08	0.141	36.24	14.130	2.05	1.46	0.35	1.74	2.86	4.71	7.75	9.70	7.96	4.89	1.78	5.19	9.25
RuSP	44	57	6.75	0.148	35.24	14.815	1.96	1.38	0.38	1.69	2.71	4.43	7.00	9.20	7.51	4.29	1.65	5.19	8.54
RuSP	44	57	7.58	0.132	43.36	13.187	1.82	1.73	0.29	1.80	3.00	5.67	8.60	10.70	8.90	5.60	1.65	4.44	7.32
RuSP	37	48	8.10	0.123	41.74	12.346	1.42	1.85	0.22	2.00	3.50	6.00	8.75	12.67	10.67	5.25	1.37	3.24	4.45
CW24h	37	48	6.80	0.147	30.85	14.706	1.59	1.36	0.36	1.80	3.00	4.89	6.88	9.80	8.00	3.88	1.37	4.17	5.72
CW24h	37	48	6.20	0.161	33.83	16.129	1.74	1.16	0.49	1.57	2.43	3.86	5.75	9.00	7.43	3.32	1.37	5.19	7.11
CW24h	56	71	7.67	0.130	40.85	13.043	2.24	1.75	0.29	1.86	3.14	5.38	8.25	10.50	8.64	5.11	2.06	5.19	10.67
CW24h	48	62	8.62	0.116	40.64	11.607	1.74	1.88	0.23	2.30	4.25	6.50	9.13	12.40	10.10	4.88	1.78	4.17	7.43
CW48h	33	43	7.44	0.134	47.54	13.433	1.41	1.66	0.31	1.72	2.80	4.60	7.63	11.40	9.68	4.83	1.23	3.70	4.57
CW48h	48	62	7.38	0.135	43.72	13.542	2.02	1.67	0.32	1.74	2.86	4.71	7.75	10.55	8.81	4.89	1.78	5.19	9.25
CW48h	67	86	7.17	0.140	34.60	13.953	2.79	1.50	0.34	1.80	3.00	5.50	7.70	9.65	7.85	4.70	2.47	6.67	16.46
CW48h	56	71	7.40	0.135	31.40	13.514	2.22	1.52	0.31	2.00	3.50	5.75	7.63	9.50	7.50	4.13	2.06	5.56	11.43
HW65d	48	62	7.15	0.140	45.95	13.978	2.10	1.55	0.38	1.65	2.63	4.25	7.63	10.55	8.90	5.00	1.78	5.93	10.57
HW65d	44	57	7.00	0.143	33.36	14.286	1.88	1.46	0.33	1.80	3.00	5.20	7.25	9.20	7.40	4.25	1.65	4.63	7.62
HW65d	44	57	7.33	0.136	42.46	13.636	1.86	1.63	0.32	1.80	3.00	5.20	7.25	10.40	8.60	4.25	1.65	4.63	7.62
HW65d	44	57	7.33	0.136	45.91	13.636	1.90	1.61	0.35	1.69	2.71	4.43	8.00	10.70	9.01	5.29	1.65	5.19	8.54
HW75d	30	38	8.50	0.118	42.65	11.765	1.10	1.91	0.18	2.07	3.67	6.50	9.50	11.80	9.73	5.83	1.10	2.36	2.59
HW75d	33	43	8.44	0.118	40.21	11.842	1.22	1.89	0.19	2.20	4.00	6.50	9.13	11.40	9.20	5.13	1.23	2.78	3.43
HW75d	44	57	8.92	0.112	41.54	11.215	1.58	1.96	0.20	2.20	4.00	7.00	10.00	12.60	10.40	6.00	1.65	3.37	5.54
HW75d	30	38	8.50	0.118	42.65	11.765	1.10	1.91	0.18	2.07	3.67	6.50	9.50	11.80	9.73	5.83	1.10	2.36	2.59

98HSO10m	59	76	8.13	0.123	41.40	12.308	2.27	1.81	0.27	2.07	3.67	6.00	9.50	11.80	9.73	5.83	2.19	5.56	12.19
98HSO10m	56	71	8.33	0.120	40.48	12.000	2.07	1.83	0.26	2.20	4.00	6.25	8.38	12.00	9.80	4.38	2.06	5.09	10.48
98HSO10m	30	38	8.50	0.118	42.65	11.765	1.10	1.91	0.18	2.07	3.67	6.50	9.50	11.80	9.73	5.83	1.10	2.36	2.59
98HSO10m	33	43	8.44	0.118	40.21	11.842	1.22	1.89	0.19	2.20	4.00	6.50	9.13	11.40	9.20	5.13	1.23	2.78	3.43
98HSO20m	44	57	7.58	0.132	39.94	13.187	1.78	1.65	0.30	1.96	3.40	5.60	7.40	10.40	8.44	4.00	1.65	4.63	7.62
98HSO20m	37	48	8.10	0.123	41.74	12.346	1.42	1.85	0.22	2.00	3.50	6.00	8.75	12.67	10.67	5.25	1.37	3.24	4.45
98HSO20m	33	43	8.44	0.118	40.21	11.842	1.22	1.89	0.19	2.20	4.00	6.50	9.13	11.40	9.20	5.13	1.23	2.78	3.43
98HSO20m	33	43	8.44	0.118	40.21	11.842	1.22	1.89	0.19	2.20	4.00	6.50	9.13	11.40	9.20	5.13	1.23	2.78	3.43

Key

**Parameter**	**Parameter description**	**Unit**
GP	Germination percentage	%
R	Relativized percentage	%
MGT	Mean germination time	day
MGR	Mean germination rate	day^{-1}
CV_t	Coefficient of variation of germination time	%, seed day^{-1}
CVG	Coefficient of velocity of germination	%
GI	Germination index	day
U	Uncertainity of germination process	bit
Z	Synchronization index	unit less
T_{10}	Time to 10% germination	day or hour
T_{25}	Time to 25% germination	day or hour
T_{50}	Time to 50% germination	day or hour
T_{75}	Time to 75% germination	day or hour
T_{90}	Time to 90% germination	day or hour
T_{10-90}	Time from 10 to 90% germination	day or hour
T_{25-75}	Time from 25 to 75% germination	day or hour
MGD	Mean daily germination Percent	%
Peak value	Peak value for germination	day^{-1} or $hour^{-1}$
G value	Germination value	
